# Vibrational Spectra of the “One-Mode” (Y_1−*x*_La*_x_*)_2_O_3_ Solid Solution Ceramics

**DOI:** 10.3390/ma19061119

**Published:** 2026-03-13

**Authors:** Xiao-Yong Zhang, Wen-Hua Shu, Dong-Yun Gui

**Affiliations:** 1 Institute of Functional Materials, College of Materials Science and Engineering, Xi′an University of Architecture and Technology, Xi’an 710055, China; 2 Zhejiang Zhiyuan Engineering Management Co., Ltd., Hangzhou 311100, China; 3 State Key Laboratory of Solidification Processing, Northwestern Polytechnical University, Xi’an 710072, China

**Keywords:** yttria compounds, Raman spectra, infrared reflection spectra

## Abstract

Y_2_O_3_ is widely used in IR windows and optoelectronics, but its vibrational and spectral properties under La^3+^ substitutions remain unclear. This work investigates *x*La_2_O_3_-(1−*x*)Y_2_O_3_ with *x* = 0–0.2 via XRD, SEM, Raman, and IR spectroscopy to address the lack of comprehensive data on structure–property correlations. The solid solution (Y_1−*x*_La*_x_*)_2_O_3_ with 0 ≤ *x* < 0.15 was determined. The cell parameter *a* increases from 10.6113(5) Å to 10.7116(1) Å as *x* increases from 0 to 0.15 according to the Rietveld refinements. Both the Raman spectra and infrared (IR) reflection spectra show that (Y_1−*x*_La*_x_*)_2_O_3_ is a “one-mode” system, in which eight of the 22 theoretical first-order Raman modes and 12 of 16 theoretical IR modes for the (Y_1−*x*_La*_x_*)_2_O_3_ are recognized. A local vibrational mode at approximately 680 cm^−1^ is observed in the Raman spectra as *x* > 0. The Raman modes and IR modes of (Y_1−*x*_La*_x_*)_2_O_3_ show red shift and obvious peak broadening as *x* increases, which is caused by the expansion of unit cell and local distortions of the crystal structure. As Y^3+^ is substituted by La^3+^, the extinction coefficient κ decreases significantly, which can lead to a lower IR absorption and should be beneficial for the IR window applications.

## 1. Introduction

Yttria (Y_2_O_3_) has been widely studied due to its extensive applications. These include infrared windows, solid-state lasers, transparent ceramics, infrared detectors, luminescent materials, and transparent optoelectronic ceramics [1,2,3,4,5,6].

The sintering of Y_2_O_3_ for a dense ceramic is challenging. Research has shown that adding small amounts of lanthanum ions (${\mathrm{La}}^{3+}$) can significantly improve the sintering of Y_2_O_3_ and the transparency in the near-to-mid-infrared range [7]. For example, Li et al. [8] obtained ${\mathrm{La}}^{3+}$-doped Y_2_O_3_ transparent ceramics and found that the precursors like La(OH)_3_ and La_2_O_2_CO_3_ improved both the sintering process and the optical transparency. Zhang et al. [1] investigated the doping effects of ${\mathrm{La}}^{3+}$ on the densification of Y_2_O_3_ under vacuum and found that the La^3+^ enhances the movement of grain boundaries—a key step for Y_2_O_3_ sintering in the presence of La^3+^. Additionally, the size difference between ${\mathrm{La}}^{3+}$ and Y^3+^ ions induces the stress within the structure, which in turn promotes the migration of Y^3+^ in the Y_2_O_3_ lattice. Yavetskiy et al. [9] also reported that ${\mathrm{La}}^{3+}$ doping improves the grain boundary movement and sintering of Y_2_O_3_ ceramics attributed to slight distortions in the crystal structure caused by the difference in ionic radius between ${\mathrm{La}}^{3+}$ and $Y^{3+}$.

Regarding the assignment of Raman active modes, only a few scientific papers have been published [10,11,12]. Zhu et al. [13] investigated the effect of ${\mathrm{Nd}}^{3+}$ doping on Y_2_O_3_, observing a red shift in the Raman bands and a significant enhancement of the 380 nm cathodoluminescence band related to oxygen vacancy. These changes were attributed to lattice distortion induced by the incorporation of the dopant ions and variations in effective absorption coefficients caused by different dopants. After Y_2_O_3_ is doped with ${\mathrm{La}}^{3+}$, its structure deviates from the ideal symmetrical structure of pure Y_2_O_3_, making vibrational mode analysis particularly challenging. Currently, there are very limited studies focusing on the theoretical calculations and vibrational spectroscopy of the (Y_1−*x*_La*_x_*)_2_O_3_ system.

Solid solution engineering is an important approach for designing and modifying materials. Investigating the phonon and lattice dynamics of solid solutions provides valuable insights into their fundamental properties [14,15,16]. The *x*La_2_O_3_-(1−*x*)Y_2_O_3_ system serves as an ideal model for studying phonon variations in solid solutions due to the systematic substitution of $Y^{3+}$ by ${\mathrm{La}}^{3+}$. In 2021, we conducted a study on the vibrational spectra and dynamic characteristics of Y_2_O_3_ through a combination of density functional theory (DFT) calculations and spectroscopic analysis [17]. While ${\mathrm{La}}^{3+}$ doping enhances sintering in Y_2_O_3_, prior studies have not comprehensively analyzed how these structural modifications affect vibrational modes or their implications for IR performance. To our knowledge, no prior research has reported similar comprehensive findings for the Y_2_O_3_-La_2_O_3_ solid solution. This study bridges this gap by combining XRD, SEM, Raman, and IR spectroscopy with group theory to clarify the relationship between La^3+^ substitution, lattice dynamics, and optical properties. Analyzing the vibrational modes of *x*La_2_O_3_-(1−*x*)Y_2_O_3_ improves our understanding of lattice dynamics and structural evolution induced by La^3+^ incorporation.

In this work, we aimed to systematically investigate the ceramic sintering mechanism, optimize the preparation process, and enhance the material performance of *x*La_2_O_3_-(1−*x*)Y_2_O_3_. Specifically, *x*La_2_O_3_-(1−*x*)Y_2_O_3_ were synthesized with 1 wt.% ZnO as an auxiliary agent. The microstructure and phase composition of the synthesized samples were thoroughly characterized. Furthermore, the infrared reflection spectra and Raman spectra were analyzed and fitted to identify characteristic vibrational modes. This study further explores the impact of La^3+^ doping on the vibrational properties of Y_2_O_3_ ceramics, thereby providing valuable insights into the underlying structural changes and densification mechanisms.

## 2. Materials and Methods

Powder samples with varying ${\mathrm{La}}^{3+}$ were synthesized using the high temperature solid-state reaction method. High-purity Y_2_O_3_ and La_2_O_3_ powders (4 N, Shanghai Macklin Biochemical Co., Ltd., Shanghai, China) were used as raw materials, weighed according to the stoichiometric ratio of (Y_1−*x*_La*_x_*)_2_O_3_ (where *x* = 0.01, 0.03, 0.05, 0.07, 0.10, 0.15, 0.20). The mixtures were ball-milled in ethanol for 6 h, followed by drying at 80 °C for 12 h. The dried powders were then calcined at 1200 °C for 24 h and subsequently sintered at 1400–1500 °C for 2 h. 1% wt. ZnO powder (3 N, Sinopharm Chemical Reagent Co., Ltd., Shanghai, China) was added as a sintering aid, and it was confirmed to be completely evaporated according to the mass loss. The sintered ceramics are grounded and polished to facilitate further characterization.

The X-ray diffraction (XRD) data were collected using a Rigaku miniFlex 600 diffractometer (Tokyo, Japan) with Cu-Kα radiation. Crystal structure analysis was conducted via Rietveld refinement. Microstructure observations of the Y_2_O_3_ ceramic samples were performed using a Tescan Vega3 (Brno, Czech Republic) for scanning electron microscopy (SEM). Raman spectra were recorded using a Renishaw inVia Raman spectrometer (Wotton-under-Edge, UK) with 633 nm lasers, achieving a spectral resolution of approximately 2 cm^−1^. Infrared reflection (IR) spectra were measured at room temperature using a Bruker 66 V Fourier transform infrared (FTIR) spectrometer (Ettlingen, Germany), covering the 50–5000 cm^−1^ range with a resolution of about 1 cm^−1^). Each IR reflectance spectrum was merged from two measurements: 50–700 cm^−1^ (far infrared, FIR) and 400–5000 cm^−1^ (medium infrared, MIR). Spectrum fitting was performed using the FOCUS-2 program developed by Meneses [18].

## 3. Results and Discussion

### 3.1. Sample and Crystal Structure

The sesquioxide Y_2_O_3_ adopts a body-centered cubic lattice, known as the bixbyite (C-type) phase, with the space group $\mathrm{Ia}\bar{3}$ (No. 206, Th^7^) under ambient conditions.

**Figure 1:**
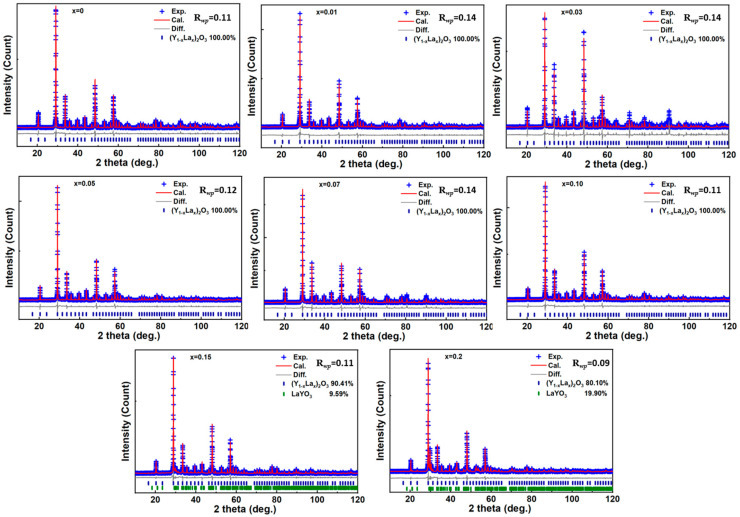
Observed XRD data and calculated profile from Rietveld refinements of *x*La_2_O_3_-(1−*x*)Y_2_O_3_ ceramics: blue cross, observed; red solid line, calculated curve; gray line below, difference curve; and vertical bars, the Bragg peak positions.

**Figure 2:**
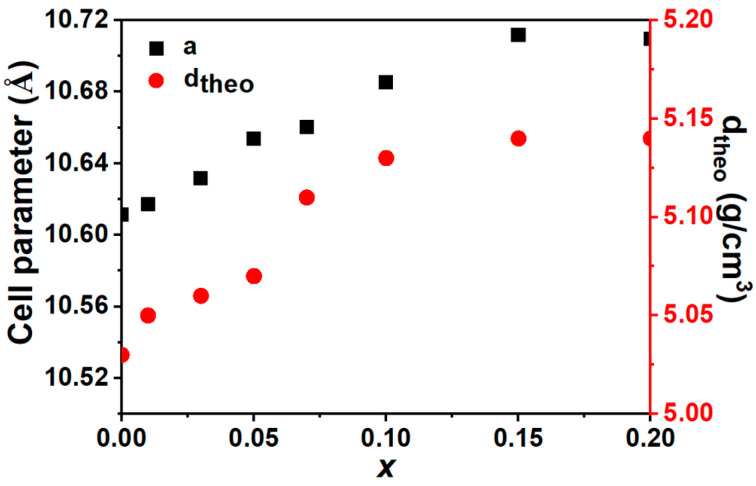
The cell parameters, *a*, and theoretical densities (*d*_theo_) of *x*La_2_O_3_-(1−*x*)Y_2_O_3_ ceramics.

The X-ray diffraction (XRD) pattern and corresponding Rietveld refinement profile for the *x*La_2_O_3_-(1−*x*)Y_2_O_3_ ceramic sample are presented in Figure 1. The strong peaks of all ceramic samples can be indexed to Y_2_O_3_ (JCPDS#83-0927). XRD analysis confirms the formation of a solid solution for (Y_1−*x*_La*_x_*)_2_O_3_ in 0 ≤ *x* < 0.15. For compositions with *x* ≥ 0.15, additional diffraction peaks are detected, which suggests the formation of secondary phases of LaYO_3_. With the increasing of La^3+^, a slight shift in the diffraction peaks toward lower angles is observed. This shift indicates an expansion of the unit cell. The cell parameter *a* and theoretical density (*d*_theo_) obtained from the Rietveld refinements is plotted in Figure 2. With the increase in La^3+^, both the *a* and *d*_theo_ increase in the solid solution region (0 ≤ *x* < 0.15) and are unchanged as *x* ≥ 0.15. The *a* and *d*_theo_ increase from 10.6113(5) Å and 5.03 (8) g/cm^3^ as *x* = 0 to 10.7116(1) Å and 5.14(2) g/cm^3^ as *x* = 0.15. In Y_2_O_3_, where $Y^{3+}$ ions are six-coordinated, the ionic radius of ${\mathrm{La}}^{3+}$ (R_La_^3+^ = 1.03 Å) is larger than that of $Y^{3+}$ (R_Y_^3+^ = 0.90 Å) [19]. Therefore, the substitution of $Y^{3+}$ by ${\mathrm{La}}^{3+}$ results in the elongation of average bond lengths, which leads to an expansion in unit cells.

### 3.2. Microstructure

**Figure 3:**
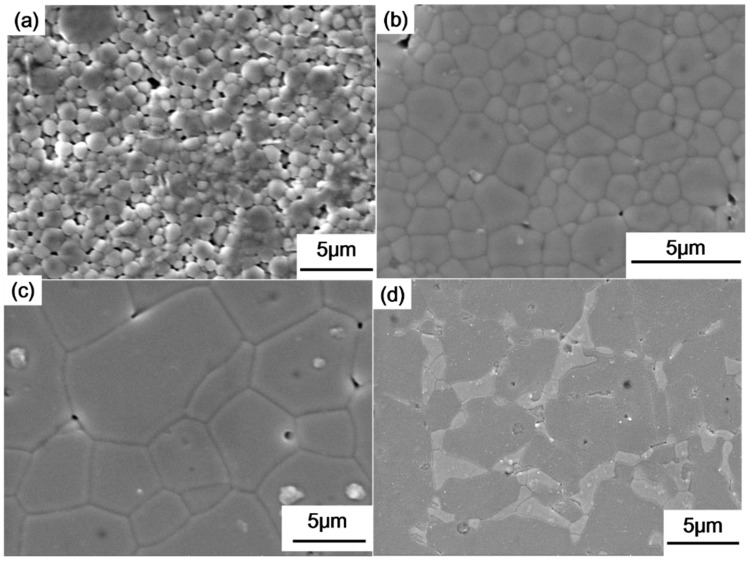
SEM images of surface morphology of *x*La_2_O_3_-(1−*x*)Y_2_O_3_ ceramics: (**a**) *x* = 0.03; (**b**) *x* = 0.05; (**c**) *x* = 0.10; and (**d**) *x* = 0.15.

Selected scanning electron microscopy (SEM) images of the fractured surface of *x*La_2_O_3_-(1−*x*)Y_2_O_3_ ceramics are presented in Figure 3. The SEM images reveal a dense and homogeneous microstructure in the ceramics. For example, the measured density of (Y_0.95_La_0.05_)_2_O_3_ is ~4.87 g/cm^3^, corresponding to a relative density of ~96%, which is consistent with the highly dense microstructure observed. No ${\mathrm{Zn}}^{2+}$ was observed in the EDS analysis due to its evaporation during the sintering. Well-crystallization, a dense microstructure and the absence of porosity ensures minimal scattering effects, which is critical for accurate Raman and IR measurements. This supports the validity of our vibrational mode assignments.

As the La_2_O_3_ concentration is increased, the average grain size is observed to gradually increase from 1 to ~10 μm. This suggests that ${\mathrm{La}}^{3+}$ accelerates mass transfer during the sintering process and enhances grain boundary mobility. This finding is consistent with the observations reported by Chen [20]. For the sample with 0.15 La_2_O_3_-0.85Y_2_O_3_ composition (Figure 3d), regions with lighter contrast are observed between the grains. These regions are attributed to the presence of a secondary phase, identified as LaYO_3_ from EDS analysis. This observation agrees with the solid solution range of 0 ≤ *x* < 0.15 for (Y_1−*x*_La*_x_*)_2_O_3_ determined from our XRD analysis.

### 3.3. Raman Spectra of xLa_2_O_3_-(1−x)Y_2_O_3_

**Figure 4:**
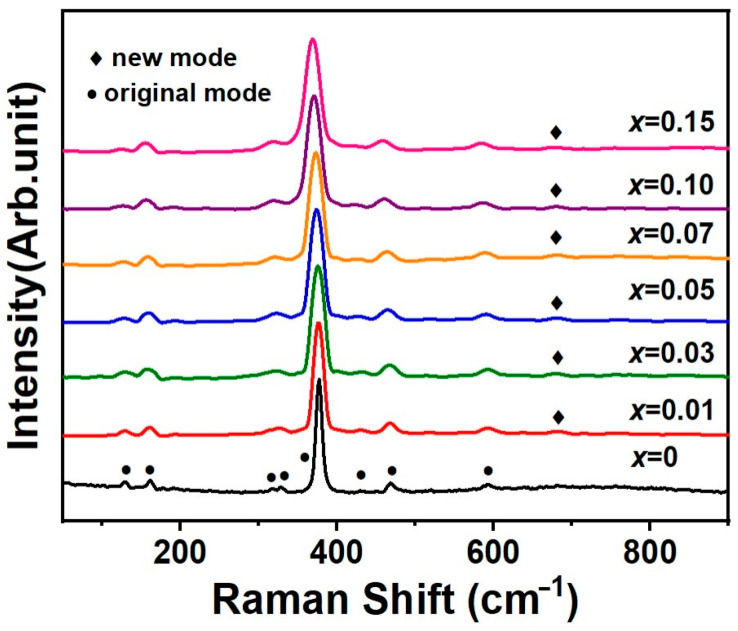
First-order Raman spectra of *x*La_2_O_3_-(1−*x*)Y_2_O_3_ ceramics.

**Figure 5:**
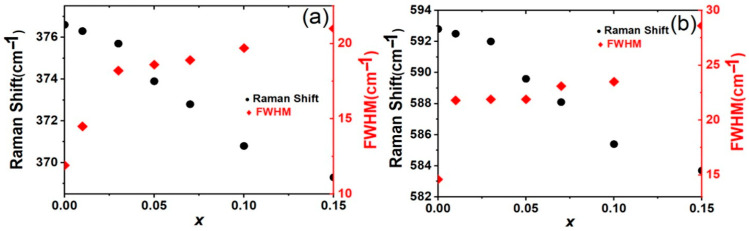
Raman shift and FWHM of *T*_g_^(9)^ (**a**) and *T*_g_^(14)^ (**b**) of *x*La_2_O_3_-(1−*x*)Y_2_O_3_ ceramics.

The Raman spectra of *x*La_2_O_3_-(1−*x*)Y_2_O_3_ are presented in Figure 4. The solid solution (Y_1−*x*_La*_x_*)_2_O_3_ shows similar Raman spectra, which agrees with the fact that the solid solution is in the same average crystal structure. According to the modified random-element-isodisplacement (MREI) model [21], (Y_1−*x*_La*_x_*)_2_O_3_ can be considered as a one-mode system, in which the frequency of the key vibrational modes shifts gradually with the change in composition (substitution concentration *x*). Eight of the 22 theoretical first-order Raman modes of Y_2_O_3_ are distinctly recognized. These Raman modes are assigned based on our previous lattice dynamics calculations employing density functional theory (DFT) [17,22]. The frequency, intensity, and peak width (full width at half maximum, FWHM) of the observed modes by peak fitting using a Voigt profile are listed in Table 1. The frequency of the vibrational modes *T*_g_^(9)^ and *T*_g_^(14)^ of different samples are shown in Figure 5. The Raman modes above 300 cm^−1^ primarily correspond to oxygen vibrations and the deformations of the rare-earth oxide (REO_6_) octahedra, where RE represents Y and La. For example, the most intense Raman band of (Y_1−*x*_La*_x_*)_2_O_3_ ceramics is centered around 376 cm^−1^, which can be attributed to the characteristic (*T*_g_^(9)^+*E*_g_^(3)^) mode of the REO_6_ octahedra. Raman modes below 200 cm^−1^ are predominantly influenced by the atomic masses of the rare-earth atoms (Y and La). A new vibrational mode at approximately 680 cm^−1^ with an FWHM of ~20 cm^−1^ is observed as *x* > 0. This mode should be the local vibrational mode (LVM), indicating localized atomic vibrations within the (Y_1−*x*_La*_x_*) _2_O_3_ solid solution lattice.

As the La^3+^ concentration increases in the (Y_1−*x*_La*_x_*)_2_O_3_, all Raman active vibrational modes are observed to broaden and gradually shift to lower frequencies. For example, the frequency of *T*_g_^(9)^ changes from 376.6 to 369.3 cm^−1^ for *x* = 0 and *x* = 0.15, and the FWHM of *T*_g_^(9)^ changes from 11.9 to 21.0 cm^−1^, respectively. The broadening of Raman peaks is attributed to a change in the local symmetry of the crystal structure, which results from local distortions caused by the ionic radii difference between the ${\mathrm{La}}^{3+}$ and $Y^{3+}$ ions. These local distortions lead to increased phonon interactions and larger vibration damping. However, diffraction-based methods (e.g., Rietveld refinements) provide an average structure and cannot directly quantify local distortions. Due to the observed peak broadening, some vibrational modes overlap and become challenging to be recognized. For instance, the FWHM of the *E*_g_^(2)^ mode is slightly larger, which is attributed to its overlap with the *T*_g_^(1)^ and *A*_g_^(1)^ modes, as well as an additional overlap between the *T*_g_^(6)^ and *E*_g_^(2)^ modes.

### 3.4. IR Reflection Spectra of xLa_2_O_3_-(1−x)Y_2_O_3_

**Figure 6:**
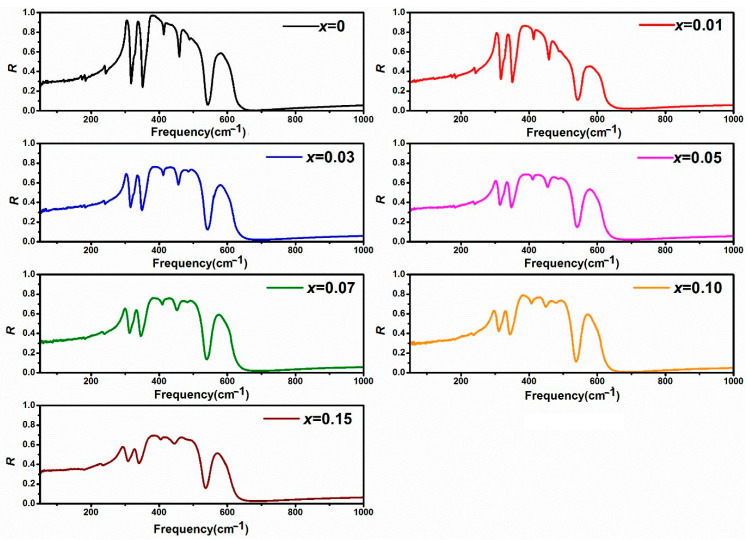
Observed infrared reflection spectrum of *x*La_2_O_3_-(1−*x*)Y_2_O_3_ ceramics.

The infrared (IR) reflection spectrum of polished (Y_1−*x*_La*_x_*)_2_O_3_ ceramics in the 50–1000 cm^−1^ is presented in Figure 6. The solid solution (Y_1−*x*_La*_x_*)_2_O_3_ shows similar IR reflection spectra as well, which agrees with the conclusion that (Y_1−*x*_La*_x_*)_2_O_3_ is a one-mode system as discussed in Section 3.3.

Vibrational modes within the material can be effectively modeled as damped oscillators. The IR reflection spectrum is often interpreted using the damped oscillator model, specifically the four-parameter semi-quantum (FPSQ) model. Within the FPSQ model [23], the frequency-dependent complex dielectric function ε*(ω) [where ε*(ω) = ε′(ω) − iε′′(ω)] is described by the following equation:

(1) $$\varepsilon^{*}\left(\omega \right)=\varepsilon_{\infty }\prod_{j=1}^{n}\frac{\Omega_{jLO}^{2}-\omega^{2}+{i\omega \gamma }_{jLO}}{\Omega_{jTO}^{2}-\omega^{2}+{i\omega \gamma }_{jTO}}$$

Here, ε_∞_ represents the high-frequency (optical) dielectric constant, which is primarily contributed by electronic polarization. *n* denotes the number of IR vibrational modes. *Ω_j_*_TO_ and *Ω_j_*_LO_ are defined as the frequencies of the *j*th transverse optical (TO) and longitudinal optical (LO) modes, respectively. Similarly, γ_jTO_ and γ_jLO_ are their corresponding damping factors.

The infrared (IR) reflection spectra of the *x*La_2_O_3_-(1−*x*)Y_2_O_3_ samples were analyzed by fitting using the four-parameter semi-quantum (FPSQ) model. A representative fitted curve is presented in Figure 6. In the 16 theoretical IR active modes, a total of 12 are distinctly resolved, with their corresponding fitted parameters listed in Table 2.

**Figure 7:**
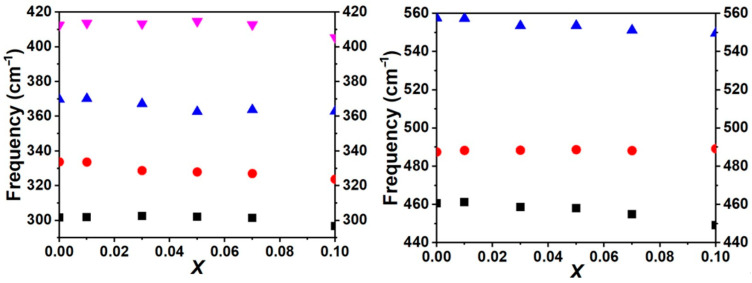
Frequency of the infrared active modes of *x*La_2_O_3_-(1−*x*)Y_2_O_3_ ceramics.

The transverse optical mode frequencies (*Ω_j_*_TO_) of some high-intensity modes show noticeable changes in Figure 7. Specifically, most of the vibrational modes are observed to shift to lower frequencies as the La concentration (*x*) increases, with this effect being particularly prominent in the sample *x* = 0.1. According to phonon characteristics of Y_2_O_3_ [17], frequencies below 239 cm^−1^ are primarily associated with the vibrations of Y-O modes. Conversely, vibrational modes with frequencies higher than 292.5 cm^−1^ are dominated by O^2−^ vibrations. As the atomic mass of ${\mathrm{La}}^{3+}$ is larger than $Y^{3+}$ and certain of $Y^{3+}$ sites are substituted by ${\mathrm{La}}^{3+}$ in samples, the substitution leads to a red shift of the low-frequency vibrational modes. For example, the *Ω_j_*_TO_ of *T*_u_^(16)^ are 557.6, 557.4, 553.5, 553.6, 551.2, 549.7, 547.9 cm^−1^ for *x* = 0.01, 0.03, 0.05, 0.07, 0.10, 0.15, and 0.20, respectively.

**Figure 8:**
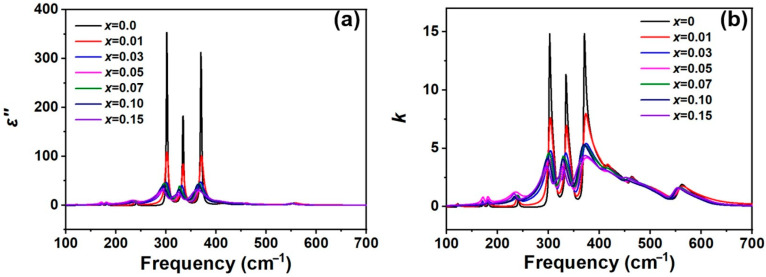
(**a**) Imaginary part of complex permittivity of *x*La_2_O_3_-(1−*x*)Y_2_O_3_ ceramics. (**b**) Extinction coefficient curve of *x*La_2_O_3_-(1−*x*)Y_2_O_3_ ceramics.

Further calculations were performed using the Fresnel equation to obtain the imaginary part of the permittivity (ε′′) and extinction coefficient curves for ${\mathrm{La}}^{3+}$-doped Y_2_O_3_ at various concentrations. These results are presented in Figure 8a and Figure 8b, respectively. The permittivity of a material is a fundamental parameter that describes the electric polarization induced by an external electric field. It is an important parameter within Maxwell’s equations and the optical properties of solid materials. The permittivity is composed of two components: the real part (ε′) and the imaginary part (ε′′). The imaginary part indicates energy dissipation due to dipole motion induced by the absorption of energy from the electric field. Both components are closely related to the extinction coefficient (*κ*) [24].

With the increase in ${\mathrm{La}}^{3+}$, both the ε′′ and *κ* curves exhibit a significant decrease in intensity and peak broadening, accompanied by a gradual shift to lower frequencies as discussed. The Y_2_O_3_ samples show sharp and narrow peaks in the ε′′ curve, whereas broadened peaks are observed in the ${\mathrm{La}}^{3+}$-doped samples. The decrease in intensity and peak broadening can be attributed to the local distortions of the crystal structure. These local distortions lead to imperfect lattice vibrations, increased phonon interactions/disturbances and larger vibration damping.

For the IR window application of Y_2_O_3_, the extinction coefficient (κ) and the maximum IR phonon frequency are key parameters that determine the spectra range and absorption. For Y_2_O_3_, the maximum IR phonon frequency is ~625.2 cm^−1^ (wavelength ~16.0 μm). When $Y^{3+}$ is substituted by ${\mathrm{La}}^{3+}$, the maximum IR phonon frequency slightly shifts to a lower frequency (~618.6 cm^−1^, wavelength ~16.2 μm as *x* = 0.15), while the κ decreases significantly. This reduction in *κ* leads to a lower IR absorption, which is beneficial for the IR window applications.

In conclusion, as *x* increases, an expansion of the unit cell parameters is observed. This expansion leads to an increase in the overall unit cell and, consequently, to larger interatomic distances. The results in a weakening of the interatomic forces, which causes a red shift in vibrational frequencies in both Raman and IR modes. On the other hand, due to the difference of ionic radii between $Y^{3+}$ and ${\mathrm{La}}^{3+}$, local distortions of the crystal structure are also introduced as the $Y^{3+}$ is substituted by ${\mathrm{La}}^{3+}$. These local distortions lead to imperfect lattice vibrations, increased phonon interactions and larger vibration damping, which causes obvious peak broadening in Raman and IR (ε′′) peaks.

## 4. Conclusions

Well-sintered *x*La_2_O_3_-(1−*x*)Y_2_O_3_ ceramics were successfully prepared using 1 wt.% ZnO as a sintering aid. From the X-ray diffraction (XRD) analysis, the formation of a solid solution as 0 ≤ *x* < 0.15 is confirmed. With the increase in La^3+^, both the cell parameter and theoretical density *d*_theo_ increase in the solid solution. La^3+^ was found to be beneficial for enhancing the densification of the ceramics. The average grain size is observed to gradually increase from 1 to ~10 μm. The vibrational spectra (both Raman spectra and infrared reflection spectra) of the Y_2_O_3_ ceramic samples were analyzed, and the observed vibrational modes were assigned. All Raman active vibrational modes are observed to broaden and gradually shift to lower frequencies. The structural perturbation and distortion of local structure is identified as the broadening of Raman peaks observed. The systematic red shift and broadening of Raman/IR modes with increasing x, combined with a reduced extinction coefficient (κ), directly correlate with enhanced IR transparency. These findings provide a design strategy for optimizing Y_2_O_3_-based ceramics for IR optical applications.

## Figures and Tables

**Table 1:** Parameters of observed Raman modes of *x*La_2_O_3_-(1−*x*)Y_2_O_3_ ceramics.

	*x* = 0.00		*x* = 0.01		*x* = 0.03		*x* = 0.05		*x* = 0.07		*x* = 0.10		*x* = 0.15	
Mode	*f*_exp._ (cm^−1^)	FWHM (cm^−1^)	*f*_exp._ (cm^−1^)	FWHM (cm^−1^)	*f*_exp._ (cm^−1^)	FWHM (cm^−1^)	*f*_exp._ (cm^−1^)	FWHM (cm^−1^)	*f*_exp._ (cm^−1^)	FWHM (cm^−1^)	*f*_exp._ (cm^−1^)	FWHM (cm^−1^)	*f*_exp._ (cm^−1^)	FWHM (cm^−1^)
*T*_g_^(1)^	128.7	10.5	129.1	14.7	129.8	19.3	127.8	17.0	126.8	17.8	125.3	19.1	124.8	22.1
*A*_g_^(1)^	161.1	10.5	160.5	12.8	158.8	16.0	159.2	15.8	158.6	15.9	156.9	16.9	155.4	17.2
*T*_g_^(6)^	316.3	11.3												
*E*_g_^(2)^	328.9	11.3	324.5	30.5	324.2	31.4	323.6	30.2	321.8	31.9	319.4	28.0	317.6	29.8
*T*_g_^(9)^	376.6	11.9	376.3	14.5	375.7	18.2	373.9	18.6	372.8	18.9	370.8	19.7	369.3	21.0
*A*_g_^(3)^	430.5	7.3	429.7	17.9	427.8	28.2	424.7	26.3	422.9	25.3	420.0	29.1	416.9	32.3
*T*_g_^(12)^	468.2	14.1	467.7	16.6	467.4	20.5	464.9	20.2	463.7	20.2	460.7	22.2	458.0	25.0
*T*_g_^(14)^	592.8	14.6	592.5	21.8	592.0	21.9	589.6	21.9	588.1	23.1	585.4	23.5	583.7	28.6
					680.6	20.3	680.7	17.7	680.0	20.51	679.1	18.6	678.2	21.9

**Table 2:** Parameters of the IR modes of *x*La_2_O_3_-(1−*x*)Y_2_O_3_ ceramics.

No.	*x* = 0.00				*x* = 0.01				*x* = 0.03				*x* = 0.05				*x =* 0.07				*x* = 0.10				*x* = 0.15			
	*Ω_j_*_TO_ (cm^−1^)	*γ_j_*_TO_	*Ω_j_*_LO_ (cm^−1^)	*γ_j_*_LO_	*Ω_j_*_TO_ (cm^−1^)	*γ_j_*_TO_	*Ω_j_*_LO_ (cm^−1^)	*γ_j_*_LO_	*Ω_j_*_TO_ (cm^−1^)	*γ_j_*_TO_	*Ω_j_*_LO_ (cm^−1^)	*γ_j_*_LO_	*Ω_j_*_TO_ (cm^−1^)	*γ_j_*_TO_	*Ω_j_*_LO_ (cm^−1^)	*γ_j_*_LO_	*Ω_j_*_TO_ (cm^−1^)	*γ_j_*_TO_	*Ω_j_*_LO_ (cm^−1^)	*γ_j_*_LO_	*Ω_j_*_TO_ (cm^−1^)	*γ_j_*_TO_	*Ω_j_*_LO_ (cm^−1^)	*γ_j_*_LO_	*Ω_j_*_TO_ (cm^−1^)	*γ_j_*_TO_	*Ω_j_*_LO_ (cm^−1^)	*γ_j_*_LO_
1	121.3	2.8	121.5	3.1	120.6	8.9	121.1	8.9																				
2	172.8	2.6	173.0	2.5	170.6	6.0	171.3	5.5	170.7	3.1	171.3	3.1	171.9	7.6	172.9	6.6												
3	182.9	2.8	183.2	2.8	180.0	5.9	181.0	6.8	179.5	6.1	180.0	7.1	180.0	6.9	181.0	8.1	176.9	1.9	177.0	2.0	180.0	10.0	180.3	10.1	181.8	2.2	182.0	2.0
4	241.1	2.5	241.6	2.5	238.7	6.1	240.0	6.4	241.0	10.5	242.2	9.0	235.8	27.9	240.6	25.5	239.3	11.7	240.5	10.3	233.5	10.1	234.6	9.7	233.7	11.5	235.1	10.1
5	301.6	2.5	315.9	4.1	301.9	7.8	315.1	5.5	302.4	21.6	314.1	5.9	302.0	23.2	312.0	8.0	300.4	22.1	311.5	8.0	296.8	23.2	308.3	9.6	297.5	25.3	305.4	10.9
6	332.0	7.1	332.3	5.2	331.6	4.7	348.9	6.1	326.2	13.1	326.5	5.2	326.1	27.1	326.6	8.7	325.9	25.3	326.6	7.2	318.2	14.5	320.0	8.8	318.9	17.0	319.0	9.0
7	333.7	2.3	349.6	4.2	333.6	4.7	348.9	6.1	328.7	9.1	347.8	8.6	327.9	7.8	345.5	10.1	327.0	6.1	345.0	10.1	323.7	12.1	341.5	13.7	321.8	15.4	336.6	13.3
8	369.7	2.8	410.2	20.9	370.1	9.6	412.7	6.9	367.2	17.8	410.8	20.7	362.6	27.4	412.6	23.6	363.7	19.1	410.9	20.4	362.8	19.9	403.3	19.3	358.5	37.0	399.4	17.8
9	412.6	23.1	458.0	8.2	413.7	7.0	458.1	13.1	413.4	20.8	456.4	11.4	414.7	22.8	455.5	17.6	412.8	19.7	453.1	13.8	405.4	21.9	447.8	13.7	400.2	20.2	449.9	24.4
10	460.6	8.2	486.4	11.8	461.3	10.7	484.2	31.6	458.6	10.9	487.0	36.3	458.1	15.8	486.8	36.5	455.0	13.0	487.0	36.3	449.1	14.4	489.0	33.3	451.5	20.5	473.1	50.3
11	487.5	12.6	535.4	17.5	488.3	35	536.8	25.6	488.4	33.3	535.3	17.1	488.7	33.0	535.6	22.5	488.2	33.2	533.2	17.6	489.2	29.6	530.8	17.7	476.3	48.3	531.5	21.0
12	557.6	14.9	620.6	32.6	557.4	19.5	624.3	63.5	553.5	17.4	621.2	30.4	553.6	19.4	622.4	38.8	551.2	17.8	619.9	28.2	549.7	16.6	614.3	28.2	547.9	19.1	618.6	41.8

## Data Availability

The original contributions presented in this study are included in the article. Further inquiries can be directed to the corresponding author.
